# Supplementary material for: Machine learning-based predictive model for prevention of metabolic syndrome
Source: PLoS One. 2023 Jun 2;18(6):e0286635. doi: 10.1371/journal.pone.0286635 (PMC10237504; doi:10.1371/journal.pone.0286635)

**Supplementary Fig. S1.** **MetS risk map with raw values.** The WC axis, representing waist circumference, displays distinct values based on gender (M for males and F for females). The BP axis transforms systolic (S) and diastolic (D) blood pressure values into equivalent values between 0 and 1, respectively, and ultimately selects the larger of the two. The raw values displayed in S, D, M, and F are rounded to one decimal place, which corresponds to the values transformed to a range between 0 and 1. As an example, for females with a waist circumference of 83 cm, systolic blood pressure of 131 mmHg, and diastolic blood pressure of 88 mmHg, the corresponding values for waist circumference, systolic blood pressure, and diastolic blood pressure are 0.4, 0.6, and 0.7, respectively, and the final blood pressure value is 0.7.


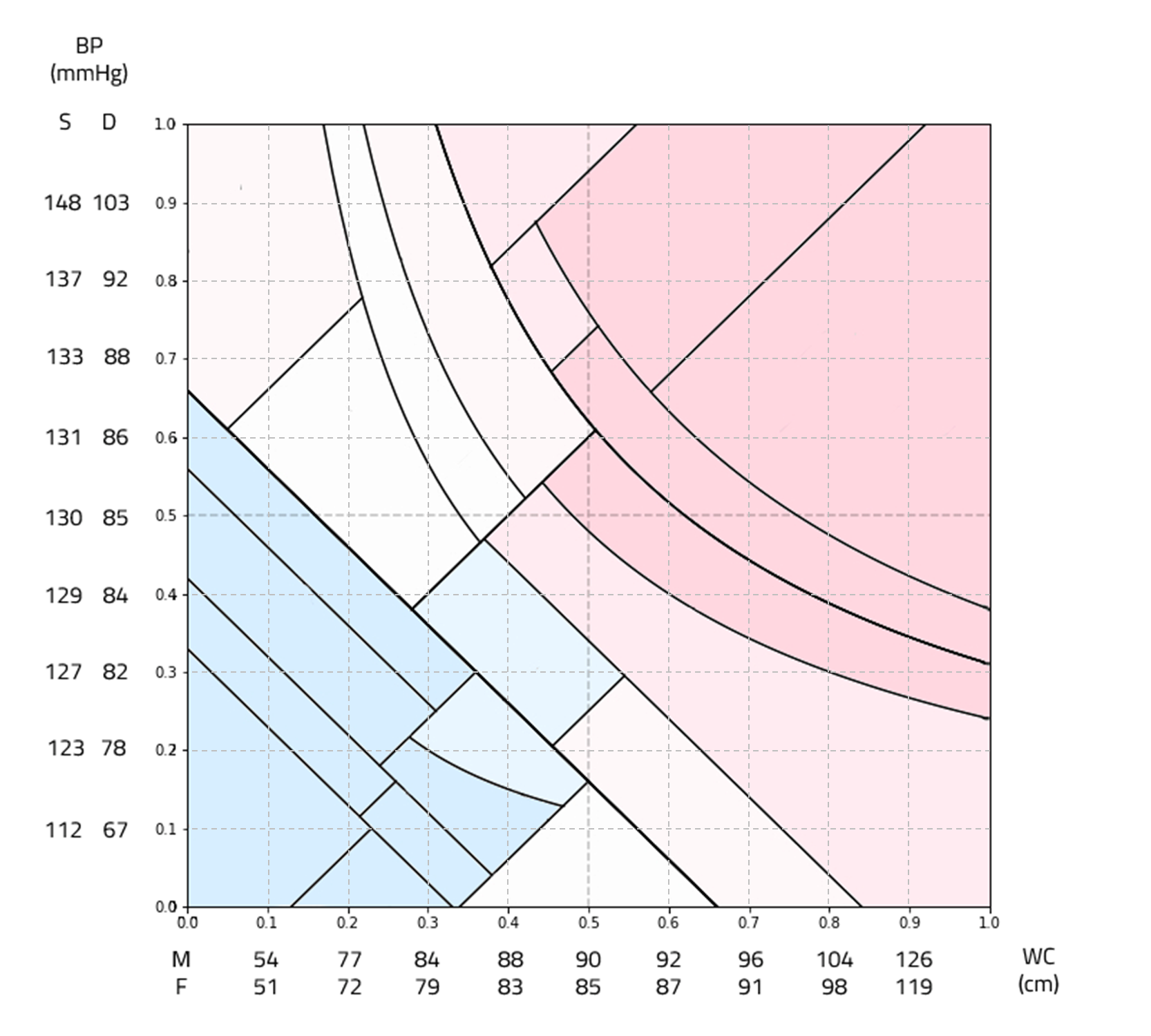

Supplement: S1 Fig — The WC axis, representing waist circumference, displays distinct values based on gender (M for males and F for females). The BP axis transforms systolic (S) and diastolic (D) blood pressure values into equivalent values between 0 and 1, respectively, and ultimately selects the larger of the two. The raw values displayed in S, D, M, and F are rounded to one decimal place, which corresponds to the values transformed to a range between 0 and 1. As an example, for females with a waist circumference of 83 cm, systolic blood pressure of 131 mmHg, and diastolic blood pressure of 88 mmHg, the corresponding values for waist circumference, systolic blood pressure, and diastolic blood pressure are 0.4, 0.6, and 0.7, respectively, and the final blood pressure value is 0.7. (DOCX) [file pone.0286635.s001.docx]
