# Supplementary figures and images for: Machine learning-based predictive model for prevention of metabolic syndrome
Source: PLoS One. 2023 Jun 2;18(6):e0286635. doi: 10.1371/journal.pone.0286635 (PMC10237504; doi:10.1371/journal.pone.0286635)

**Supplementary Fig. S2. An example of a region being divided into decision boundaries.**


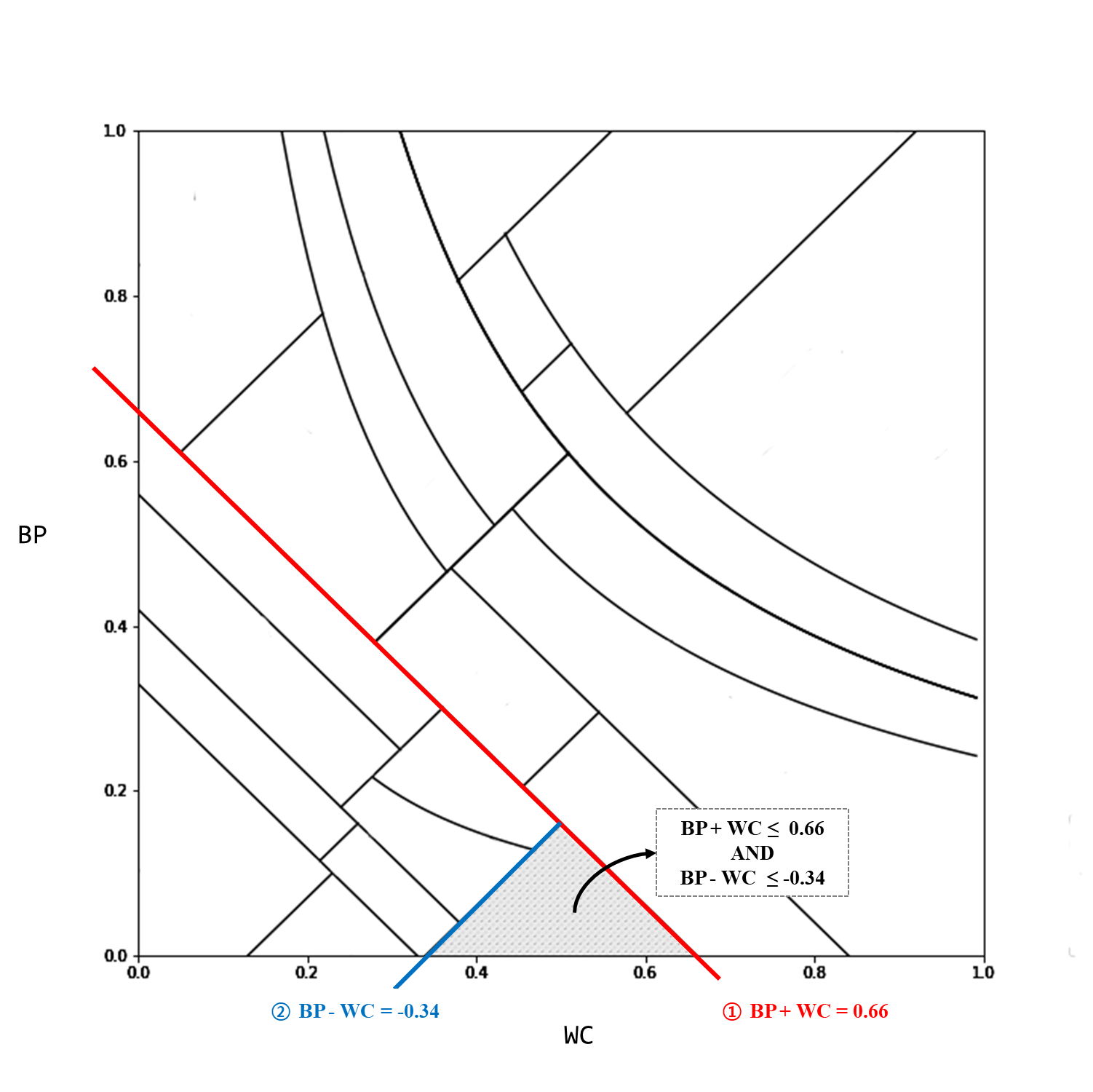

Supplement: S2 Fig — (DOCX) [file pone.0286635.s002.docx]
