# Supplementary material for: Machine learning-based predictive model for prevention of metabolic syndrome
Source: PLoS One. 2023 Jun 2;18(6):e0286635. doi: 10.1371/journal.pone.0286635 (PMC10237504; doi:10.1371/journal.pone.0286635)
Supplement: S1 Table — The ruleset on the left of the decision tree is the final model that was used to construct the risk map in this study. The ruleset on the right is a reconstruction of the left ruleset (BPWC_add, BPWC_dif, BPWC_mul) into BP and WC. Each rule serves as a decision boundary that divides a two-dimensional plane. For instance, the first two rule sets, BP + WC < = 0.66 and BP—WC < = -0.34, form two decision boundaries (red and blue) in S2 Fig, resulting in a gray area. (DOCX) [file pone.0286635.s003.docx]

**Supplementary Table. S1.** **Structure of the final decision tree.** The ruleset on the left of the decision tree is the final model that was used to construct the risk map in this study. The ruleset on the right is a reconstruction of the left ruleset (BPWC_add, BPWC_dif, BPWC_mul) into BP and WC. Each rule serves as a decision boundary that divides a two-dimensional plane. For instance, the first two rule sets, BP + WC <= 0.66 and BP - WC <= -0.34, form two decision boundaries (red and blue) in Fig. S2, resulting in a gray area.

| \|--- BPWC_add <= 0.66  \| \|--- BPWC_dif <= -0.34  \| \| \|--- class: 0  \| \|--- BPWC_dif > -0.34  \| \| \|--- BPWC_add <= 0.42  \| \| \| \|--- BPWC_add <= 0.33  \| \| \| \| \|--- BPWC_dif <= -0.13  \| \| \| \| \| \|--- class: 0  \| \| \| \| \|--- BPWC_dif > -0.13  \| \| \| \| \| \|--- class: 0  \| \| \| \|--- BPWC_add > 0.33  \| \| \| \| \|--- BPWC_dif <= -0.10  \| \| \| \| \| \|--- class: 0  \| \| \| \| \|--- BPWC_dif > -0.10  \| \| \| \| \| \|--- class: 0  \| \| \|--- BPWC_add > 0.42  \| \| \| \|--- BPWC_dif <= -0.06  \| \| \| \| \|--- BPWC_mul <= 0.06  \| \| \| \| \| \|--- class: 0  \| \| \| \| \|--- BPWC_mul > 0.06  \| \| \| \| \| \|--- class: 0  \| \| \| \|--- BPWC_dif > -0.06  \| \| \| \| \|--- BPWC_add <= 0.56  \| \| \| \| \| \|--- class: 0  \| \| \| \| \|--- BPWC_add > 0.56  \| \| \| \| \| \|--- class: 0  \|--- BPWC_add > 0.66  \| \|--- BPWC_mul <= 0.31  \| \| \|--- BPWC_dif <= 0.10  \| \| \| \|--- BPWC_add <= 0.84  \| \| \| \| \|--- BPWC_dif <= -0.25  \| \| \| \| \| \|--- class: 1  \| \| \| \| \|--- BPWC_dif > -0.25  \| \| \| \| \| \|--- class: 0  \| \| \| \|--- BPWC_add > 0.84  \| \| \| \| \|--- BPWC_mul <= 0.24  \| \| \| \| \| \|--- class: 1  \| \| \| \| \|--- BPWC_mul > 0.24  \| \| \| \| \| \|--- class: 1  \| \| \|--- BPWC_dif > 0.10  \| \| \| \|--- BPWC_mul <= 0.17  \| \| \| \| \|--- BPWC_dif <= 0.56  \| \| \| \| \| \|--- class: 0  \| \| \| \| \|--- BPWC_dif > 0.56  \| \| \| \| \| \|--- class: 0  \| \| \| \|--- BPWC_mul > 0.17  \| \| \| \| \|--- BPWC_mul <= 0.22  \| \| \| \| \| \|--- class: 1  \| \| \| \| \|--- BPWC_mul > 0.22  \| \| \| \| \| \|--- class: 1  \| \|--- BPWC_mul > 0.31  \| \| \|--- BPWC_dif <= 0.44  \| \| \| \|--- BPWC_mul <= 0.38  \| \| \| \| \|--- BPWC_dif <= 0.23  \| \| \| \| \| \|--- class: 1  \| \| \| \| \|--- BPWC_dif > 0.23  \| \| \| \| \| \|--- class: 1  \| \| \| \|--- BPWC_mul > 0.38  \| \| \| \| \|--- BPWC_dif <= 0.08  \| \| \| \| \| \|--- class: 1  \| \| \| \| \|--- BPWC_dif > 0.08  \| \| \| \| \| \|--- class: 1  \| \| \|--- BPWC_dif > 0.44  \| \| \| \|--- class: 1 | \|--- BP + WC <= 0.66  \| \|--- BP - WC <= -0.34  \| \| \|--- class: 0  \| \|--- BP - WC > -0.34  \| \| \|--- BP + WC <= 0.42  \| \| \| \|--- BP + WC <= 0.33  \| \| \| \| \|--- BP - WC <= -0.13  \| \| \| \| \| \|--- class: 0  \| \| \| \| \|--- BP - WC > -0.13  \| \| \| \| \| \|--- class: 0  \| \| \| \|--- BP + WC > 0.33  \| \| \| \| \|--- BP - WC <= -0.10  \| \| \| \| \| \|--- class: 0  \| \| \| \| \|--- BP - WC > -0.10  \| \| \| \| \| \|--- class: 0  \| \| \|--- BP + WC > 0.42  \| \| \| \|--- BP - WC <= -0.06  \| \| \| \| \|--- BP * WC <= 0.06  \| \| \| \| \| \|--- class: 0  \| \| \| \| \|--- BP * WC > 0.06  \| \| \| \| \| \|--- class: 0  \| \| \| \|--- BP - WC > -0.06  \| \| \| \| \|--- BP + WC <= 0.56  \| \| \| \| \| \|--- class: 0  \| \| \| \| \|--- BP + WC > 0.56  \| \| \| \| \| \|--- class: 0  \|--- BP + WC > 0.66  \| \|--- BP * WC <= 0.31  \| \| \|--- BP - WC <= 0.10  \| \| \| \|--- BP + WC <= 0.84  \| \| \| \| \|--- BP - WC <= -0.25  \| \| \| \| \| \|--- class: 1  \| \| \| \| \|--- BP - WC > -0.25  \| \| \| \| \| \|--- class: 0  \| \| \| \|--- BP + WC > 0.84  \| \| \| \| \|--- BP * WC <= 0.24  \| \| \| \| \| \|--- class: 1  \| \| \| \| \|--- BP * WC > 0.24  \| \| \| \| \| \|--- class: 1  \| \| \|--- BP - WC > 0.10  \| \| \| \|--- BP * WC <= 0.17  \| \| \| \| \|--- BP - WC <= 0.56  \| \| \| \| \| \|--- class: 0  \| \| \| \| \|--- BP - WC > 0.56  \| \| \| \| \| \|--- class: 0  \| \| \| \|--- BP * WC > 0.17  \| \| \| \| \|--- BP * WC <= 0.22  \| \| \| \| \| \|--- class: 1  \| \| \| \| \|--- BP * WC > 0.22  \| \| \| \| \| \|--- class: 1  \| \|--- BP * WC > 0.31  \| \| \|--- BP - WC <= 0.44  \| \| \| \|--- BP * WC <= 0.38  \| \| \| \| \|--- BP - WC <= 0.23  \| \| \| \| \| \|--- class: 1  \| \| \| \| \|--- BP - WC > 0.23  \| \| \| \| \| \|--- class: 1  \| \| \| \|--- BP * WC > 0.38  \| \| \| \| \|--- BP - WC <= 0.08  \| \| \| \| \| \|--- class: 1  \| \| \| \| \|--- BP - WC > 0.08  \| \| \| \| \| \|--- class: 1  \| \| \|--- BP - WC > 0.44  \| \| \| \|--- class: 1 |
| --- | --- |
