# Supplementary material for: Machine learning-based predictive model for prevention of metabolic syndrome
Source: PLoS One. 2023 Jun 2;18(6):e0286635. doi: 10.1371/journal.pone.0286635 (PMC10237504; doi:10.1371/journal.pone.0286635)
Supplement: Attachment — Submitted filename: comments.pdf [file pone.0286635.s005.pdf]

Thanks to the authors for the edits made to the manuscript based on my previous comments. I still have few minor comments I would like the authors to address:

1) The MetS risk map in Fig. 9 showed two boundaries, left diagonal line (WC + BP) and right curve (WC * PB), for patients at risk of MetS. This dividing line and curve were drawn on the basis of the synthetic variables WC and BP. Although the authors gave values for WC and BP indicating boundary crossing in their discussion, is it possible to provide raw values of waist circumference, systolic blood pressure, diastolic blood pressure and sex for the boundary line and the curve? Then compare the raw values of wc, sbp, dbp, and sex to the Korean diagnostic criteria for MetS that you presented in the "Preprocessing and Feature Summary" section? By this comment I would like to know, for example, how far or close the raw male/female wc are to risk factors for wc (>=90cm/>=85cm).

2) Can the authors provide the script used to generate the risk maps in their shared github repository?

3) Although high triglycerides and reduced HDL cholesterol are risk factors for MetS, none of the ML algorithms selected for these two variables. Is it possible that the authors discuss why they are not selected, is it because these variables have less impact on the early prediction of MetS or is it specific to the Korean population?

4) Typographical error in line 76, “of our”

5) Line 100, 101, eight cities and eight provinces were provided, not nine.
