# Supplementary material for: Machine learning-based predictive model for prevention of metabolic syndrome
Source: PLoS One. 2023 Jun 2;18(6):e0286635. doi: 10.1371/journal.pone.0286635 (PMC10237504; doi:10.1371/journal.pone.0286635)
Supplement: Attachment — Submitted filename: Response_to_reviewers.docx [file pone.0286635.s004.docx]

Response to reviewers

**Reviewer #1**

**Major concerns:**

1. Based on the large sample size (n=70,370) it can be expected that the different ML algorithms will exhibit comparative performance metrics. In addition, we can already see in Table 1 that DT in several studies used the fewest features for the prediction of MetS. It is unclear from the manuscript the reason for using five algorithms and what the criteria for selecting the algorithms are.

**Answer:**

Our objective is to develop an efficient and non-invasive metabolic syndrome prediction model that could aid in preventing metabolic disorders in the Korean population. While the decision tree (DT) model has been extensively researched, its applicability in the Korean context has been inadequately explored. Consequently, this study seeks to investigate the transferability of the previous findings on the DT model to the Korean population and propose modifications to enhance its interpretability.

The selection criteria for the five models are delineated as follows: First, the logistic regression (LR) model was chosen as a benchmark model for performance comparison. LR, a conventional algorithm alongside DT, is renowned for its interpretability. DT and TN were chosen based on their interpretive characteristics. DT possesses an innate quality of being easily comprehensible by non-experts if it has a reasonable number of nodes. TN, a recent deep learning model, can generate feature maps through a learnable mask, representing features highly correlated with prediction results. It was postulated that TN's interpretability could render it the most appropriate option if it exhibits a discernable difference in performance compared to existing models. RF and XG were selected for performance evaluation since the ensemble model RF and boosting family XG are known to be the highest-performing models in tabular data. Consequently, the performance of these models served as an upper limit to gauge the positioning of interpretable models DT and TN.

The rationale for selecting the DT-series algorithm lies in its inherent flexibility, which can be attributed to two primary characteristics. First, decision trees are categorized as non-parametric methods, thereby implying that they are not constrained by any assumptions pertaining to the distribution of the space. Second, decision trees are distance-based models that neither require normalization nor scale conversion and are robust to the presence of outliers.

As a result, we have concluded that developing practical predictive models utilizing the aforementioned algorithms is warranted. In the Classification algorithms section, the reasons for selecting the five algorithms were modified to be well revealed.

2. The authors used Feature Selection Wrapper on the five ML algorithms to select the best combination of features based on model performance neglecting the relationship between the selected features and the response variable. Thus, the use of filter-based methods for feature selection will add a statistical significance to the features identified in this study.

**Answer:**

It appears that we may have differing interpretations regarding the wrapper method. Wrapper methods account for the relationship between features and the response variable, and when applied to a decision tree model, this relationship is based on information theory. The nature of the relationship between features and the response variable may vary depending on the evaluation criteria employed, including correlation, Euclidean distance, and information measurements. Nonetheless, the wrapper method can capture the relationship between features and dependent variables. In addition to its ability to consider feature interactions and make classifier-dependent feature selections, the Wrapper method is known to exhibit high classification performance in most cases when compared to the Filter method. However, this method has the disadvantage of requiring a higher amount of computation, and there is a risk of overfitting. Notably, the results obtained using the Wrapper method in this study were marginally better than those obtained using mRMR, which is known to perform well. mRMR is a method used to select features while considering the redundancy between features and the correlation between features and the response variable. Please refer to the following papers.

<https://doi.org/10.1016/j.neucom.2017.11.077>

<https://doi.org/10.1093/bioinformatics/btm344>

3. Sex feature was among the core features used by all classifiers, hence males and females may have different risk factors for MetS. It is therefore possible, if considered separately, to identify specific features for males and females that can increase the accuracy of the ML models.

**Answer:** We concur that the predictive model’s performance or the selected features may differ based on gender. Nonetheless, the study results indicated that the gender-based model did not yield a significant improvement in performance. We described the following sentences in the discussion section:

“Of the studies listed in Table 14, two studies ([9] and [13]) identified gender-specific differences in model performance when considering prevalence-robust metrics. Specifically, male models outperformed female models in both studies, with a male balanced accuracy of 0.807 and female of 0.646 in [9] and a male recall of 0.594 and female of 0.409 in [13]. While key anthropometric features were consistent across genders, variations were observed in food-related features. Therefore, we developed the final decision tree (DT) model separately for men and women and compared their performance (Table 14): the balanced accuracy was 0.872 for men and 0.890 for women, and the recall was 0.843 for men and 0.850 for women. Both gender models shared common features such as BPWC_add and BPWC_mul (Fig 11), while the female model included additional anthropometric and dairy-related food features. However, when tested on the same dataset, the integrated model performed better for men based on AUC, while no significant difference was observed for women. Consequently, the study concludes that the individual models' impact on feature selection and performance was insignificant.”

**Fig. 11.** **Features and feature importance of integrated and sex-specific individual models.** “grp35” is a synthetic feature related to dairy products.

**Table 14. Performance of integrated and sex-specific individual models using the same test set**

| **Case** | | **AUC** | **Recall** | **Specificity** | **BACC** | **Features** |
| --- | --- | --- | --- | --- | --- | --- |
| **Integrated Model** | **All** | 0.889 | 0.855 | 0.773 | 0.814 | **BPWC_add, BPWC_mul,** BPWC_dif |
|  | **Male** | 0.878 | 0.895 | 0.690 | 0.792 |  |
|  | **Female** | 0.890 | 0.826 | 0.806 | 0.816 |  |
| **Sex-based model** | **Male** | 0.872 | 0.843 | 0.736 | 0.790 | **BPWC_add, BPWC_mul,** WC |
|  | **Female** | 0.890 | 0.850 | 0.780 | 0.815 | **BPWC_add, BPWC_mul,** bWC, CUNBAE, BFP, dairy products |

4. The positive predictive value (PPV) of the DT model, based on the confusion matrix in Figure 10, is 37%. This low value demonstrates the model's inability to predict the presence of MetS in a patient and possibly put the patient at risk associated with unnecessary heavy treatment and strict dieting. Although the authors presented a warning zone using a risk map, the boundaries of the zone are wide and can put patients at risk. Related to the previous comment, wondering what the proportions of misclassified cases are among males and females?

**Answer:** We moved the misclassification analysis in the analysis section to the discussion section and supplemented it from a PPV perspective to describe it as follows:

From a positive predictive value (PPV) perspective, it is possible to divide the risk map into three distinct areas: green, yellow, and red (Fig 12(A)). The green zone refers to a section that is not associated with MetS, where the risk of developing metabolic syndrome is 0 to 1 times or lower than the average prevalence of 13.7%. The yellow zone refers to a section in which the risk of developing MetS is 1 to 2.5 times higher. This transitional phase represents a mixed area of cases, including both non-MetS and MetS. Despite its ambiguity due to a low PPV of 0.248, it is imperative to prioritize the prevention and management of MetS in this state, given the increased risk probability compared to the average. The red zone refers to a section in which the risk of developing MetS is 4 to 5.7 times higher. With a PPV of 0.673, this area is deemed as having a high likelihood of actual metabolic syndrome cases, necessitating blood tests for accurate diagnosis.

**Fig. 12.** **Risk map from PPV perspective and the risk index distribution in FP cases** (A) Three zones of risk map from the perspective of PPV perspective: green, yellow, and red zone. (B) Risk index distribution of FP in yellow zone. (C) Risk index distribution of FP in red zone. 0.45 is the point where MetS management is required preemptively (indicated by a red dotted line).

Table 15 summarizes the positive predictive values (PPV) for each gender in different zones. The Yellow Zone indicates relatively low PPV for both males (0.258) and females (0.241), implying that only one out of four predicted individuals are likely to have metabolic syndrome. In contrast, the Red Zone exhibits higher PPV for males (0.725) and females (0.633) compared to the Yellow Zone, with males having a higher PPV. These findings suggest that 6 to 7 out of 10 predicted individuals are highly likely to have metabolic syndrome. However, the use of binary classification diagnostic criteria to calculate PPV is inadequate for prevention of metabolic syndrome, which is a chronic disease that develops progressively over time. Our focus, therefore, should be on providing individuals with opportunities to manage the condition before it progresses to a more severe stage. Thus, in this study, we analyzed FP from the perspective of severity, rather than solely on the presence of metabolic syndrome. To achieve this, we employed the risk index proposed in our previous work [18] and obtained a risk distribution for the FP cases. The resulting risk distribution is presented in Fig 12 (B-C). Our risk index employs a diagnostic threshold of 0.547 for identifying MetS [18]. However, MetS may also occur at values as low as 0.45, leading us to classify individuals in the MetS risk group as having a score of 0.45. Using this criterion, we identified 552 of the 1176 FP cases in the Yellow Zone as true positives, resulting in an adjusted PPV of 0.582, up from 0.248. Similarly, in the red zone, 123 FP cases were reclassified as true positives, resulting in an adjusted PPV of 0.686, up from 0.673. Nevertheless, to enhance PPV significantly, additional research is imperative to enhance the performance by configuring individual models for each zone or region exhibiting multiple misclassifications.

**Table 15. Misclassification by zone**

| Zone | Group | FN | FP | TN | TP | PPV | NPV | NT-> TP | Adjusted PPV |
| --- | --- | --- | --- | --- | --- | --- | --- | --- | --- |
| Green+Yellow+Red | ALL | 137 | 1382 | 4,707 | 811 | 0.370 | 0.972 | 675 | 0.678 |
|  | Male | 42 | 534 | 1,187 | 359 | 0.402 | 0.966 | 273 | 0.708 |
|  | Female | 95 | 848 | 3,520 | 452 | 0.348 | 0.974 | 302 | 0.580 |
| Green | ALL | 137 | 0 | 4,707 | 0 | - | 0.972 | - | - |
|  | Male | 42 | 0 | 1,187 | 0 | - | 0.966 | - | - |
|  | Female | 95 | 0 | 3,520 | 0 | - | 0.974 | - | - |
| Yellow | ALL | 0 | 1176 | 0 | 387 | 0.248 | - | 552 | 0.582 |
|  | Male | 0 | 458 | 0 | 159 | 0.258 | - | 229 | 0.629 |
|  | Female | 0 | 718 | 0 | 228 | 0.241 | - | 223 | 0.551 |
| Red | ALL | 0 | 206 | 0 | 424 | 0.673 | - | 123 | 0.868 |
|  | Male | 0 | 76 | 0 | 200 | 0.725 | - | 44 | 0.884 |
|  | Female | 0 | 130 | 0 | 224 | 0.633 | - | 79 | 0.856 |

FN: False Negative, FP: False Positive, TN: True Negative, TP: True Positive, NPV: Negative Predictive Value, PPV: Positive Predictive Value. ‘NP->TP’ is the number of cases that can be reclassified from NP to TP when determining whether or not to be prevented from MetS based on the risk index 0.45. Adjusted PPV is the value calculated by reflecting ‘NP->TP’.

Although there is a limit to the low PPV, our final model can effectively help the decision-making process in preventing and managing MetS by providing development risks as well as good discrimination and recall.

5. This is another study that was conducted on Korean participants (https://doi.org/10.3390/app10217741). They reported the association of triglycerides with MetS, a feature not detected by any of the ML algorithms employed in the manuscript. This study may also invalidate the authors' claim regarding the comparability of the manuscript's findings with other published studies on the fact of race. Could the authors elaborate and discuss their findings with the results presented in the link above?

**Answer:** Triglycerides are a diagnostic criterion for metabolic syndrome and can be assessed through blood tests. However, the objective of our study differs from that of the previous research (https://doi.org/10.3390/app10217741), as it aims to identify metabolic syndrome using non-invasive factors exclusively, without relying on invasive measures such as blood test results.

**Minor comments:**

1. It is not clear why the authors used Elliot sigmoid function for WC, SBP, DBP, and why they used the difference between sbp and dbp in the denominator?

**Answer:** The advantage of using a sigmoid function is that an outlier does not affect other values, and any value including the outlier can be converted to a range between 0 and 1. In addition, using a sigmoid function, the closer to the diagnostic criterion, the more sensitive the risk that can be reflected. As shown in Fig. A, as the value of the X-axis approaches zero, the amount of change on the Y-axis increases.

Furthermore, in this study, the Elliot sigmoid function was applied instead of the basic sigmoid function because the Elliott sigmoid can more sensitively reflect changes near the MetS diagnostic criteria. Fig. A shows that the Elliot sigmoid has a larger slope near zero than the basic sigmoid function.

Fig. A. Sigmoid function (purple) and Elliot sigmoid function (red)

For blood pressure, the larger of the two was selected when subtracting each threshold from systolic and diastolic blood pressure. The selected value was then adjusted such that the size of one unit was the same, the difference between the two thresholds, rather than each threshold.

In this study, synthetic feature BP represents a value that deviates more from each diagnostic criterion among both systolic and diastolic blood pressure. At this time, the difference between the two blood pressure diagnostic criteria was commonly used as one of the methods of matching the meaning of the unit value of BP. This can be interpreted as, for example, when BP is 0.75, the systolic blood pressure or diastolic blood pressure exceeds the threshold value of 4.5 $mg/dl$*:* 4.5=(130-85)*0.1.

2. The CLBE synthetic feature is not mentioned in Table 7 or any other table.

**Answer:** We corrected the error. CLBE is a carbohydrate energy feature.

3. The female WC value of 0.45 in the example was calculated based on the male diagnostic criteria of 9 rather than 8.5. The WC value should be 0.66.

**Answer:** The error was corrected by applying the female criteria. We corrected the errors in the text and Fig 7.

“… converted to 0.84 (BP) and 0.66 (WC). After that, the conversion values are once again converted to 1.50 (BPWC_add), 0.55 (BPWC_mul), and 0.18 (BPWC_dif) …”

4. The authors should share their source code in a public repository for reproducibility and for the benefit of the scientific community.

**Answer:** We released the experimental codes in the following online repository.

- <https://github.com/shinhseok/predictive-model-for-prevention-of-MetS>

**Reviewer #2**

1. The authors have acquired the medical histories of individuals. What point in time was considered for the development of the model? Was it the latest?

**Answer:** The medical history was gathered during a patient’s examination, through a questionnaire filled out by the patient. The medical history is up to date and at the time of data collection.

2. Samples were collected between 2004 and 2013. However, the analysis was conducted in 2022. Why were the post 2014 samples excluded from the analysis as you highlighted the increase in cases in 2018? If possible, it would be interesting to see model performance on post-2013 data that was trained on pre-2013 data.

**Answer:** According to the data cited in the introduction (<https://doi.org/10.51789/cmsj.2021.1.e15>), the prevalence rate of the condition in Korea has increased steadily, reaching 22.9% in 2018. However, it is important to note that even before 2013, the prevalence rate was already high, ranging from 19.4% to 21.6%. The data used in this study comes from a cohort study called KoGES_HEXA, which was conducted by the Korea Centers for Disease Control and Prevention. To date, this study has been conducted twice. The first survey, which collected data from approximately 170,000 individuals, was conducted between 2004 and 2013, while the second survey was a follow-up conducted between 2012 and 2016, which collected data from approximately 65,000 people. For this study, we primarily utilized the larger dataset obtained from the first survey.

In the discussion section, we acknowledged that the use of 2004-2013 data was a limitation, even though it would have been preferable to use the most current dataset available.

“Moreover, it should be noted that the analysis of this study is based on datasets collected from 2004-2013, so there is a time difference of more than 10 years.”

3. Did authors attempt to test performance for different combinations of feature from different group of features (anthropometric + survey-based features (given), survey-based features +synthetic features and anthropometric + synthetic features) at in round two in Figure 3?

**Answer:** The process of selecting optimal features for the study involved various attempts, including changing the order of the three feature groups, creating different feature groups, eliminating feature groups, and refining them to change the screening order between each group. Despite these efforts, no significantly better results were obtained than the final results.

4. If possible, please provide a sample distribution by year for both groups.

**Answer:** We understand that the group you mentioned refers to gender. In Table 2 and the raw data sections, we have already described the average age, standard deviation, and range (40-70 years) by gender.

5. If possible, please put your code on GitHub. It is useful to test and reuse the code.

**Answer:** We released the experimental codes in the following online repository.

- <https://github.com/shinhseok/predictive-model-for-prevention-of-MetS>

6. Is the data available in the public domain, if yes, please provide the link.

**Answer:** Access to the data used in this study is subject to conditional disclosure, in accordance with the policies of the Korea Centers for Disease Control and Prevention. Interested parties must seek permission from the Korea Centers for Disease Control and Prevention in order to access the data. Conditional disclosure data can be accessed via the application page, accessible through the following links.

- <http://www.cdc.go.kr/board.es?mid=a20602010000&bid=0034&act=view&list_no=77471>
- <https://nih.go.kr/biobank/cmm/main/engMainPage.do>

7. If the list of major cities is not big, could you please list them.

**Answer:** In the Raw Data section, we have added the following information about major cities.

“This study was based on health checkup records collected from South Korea: nine major metropolitan cities (Seoul, Incheon, Daegu, Busan, Gwangju, Ulsan, Daejeon, and Sejong) and nine other provinces (Gyeonggi-do, Gangwon-do, Gyeongsangnam-do, Chungcheongbuk-do, Chungcheongnam-do, Jeollanam-do, Jeollabuk-do, and Jeju-do).”

8. Other corrections:

**Answer:** We modified all of the following corresponding sentence expressions. We obtained additional proofread from a native speaker and have attached a certificate of proofreading.

1. Line 10-11: high blood sugar, and dyslipidemia and can lead to cardiovascular disease or type 2 diabetes

🡪 high blood sugar and dyslipidemia, and can lead to cardiovascular disease or type 2 diabetes

2. Line 31: This tool should be used to predict MetS using only noninvasive information

🡪 Delete

3. Line 38: The number of features used in the final model, counted based on unsynthesized features. 🡪 Only unsynthesized features were counted in the final model.

4. Line 59: focusing on classification performance

🡪 focusing only on classification performance

5. 62: when we try to apply predictive models from previous studies to real life

🡪 when we apply predictive models from previous studies to real life.

6. 70-71: and compared their usability. How do you compare usability? I guess you mean different parameters such as AUC, accuracy, recall etc.

… and compared their usability. 🡪 and compared their performance by AUC, sensitivity, specificity, balanced accuracy and number of features.

7. 83: All the features were arranged in tabular datasets, excluding outliers and missing values. Did you mean subject instead of feature? If not, how did you identify outlier features?

All the features were arranged in tabular datasets, excluding outliers and missing values. 🡪 After excluding subjects with outliers and missing values, all features were arranged in a tabular data set.
